# Dynamic changes in the transcriptome landscape of *Arabidopsis thaliana* in response to cold stress

**DOI:** 10.3389/fpls.2022.983460

**Published:** 2022-08-30

**Authors:** Yue Liu, Yajun Cai, Yanzhuo Li, Xiaoling Zhang, Nan Shi, Jingze Zhao, Hongchun Yang

**Affiliations:** ^1^State Key Laboratory of Hybrid Rice, College of Life Sciences, Wuhan University, Wuhan, China; ^2^Hubei Hongshan Laboratory, Wuhan, China; ^3^RNA Institute, Wuhan University, Wuhan, China

**Keywords:** *Arabidopsis thaliana*, cold stress, ssRNA-seq, transcriptome landscape, dynamic change, long non-coding RNA, alternative splicing

## Abstract

Plants must reprogram gene expression to adapt constantly changing environmental temperatures. With the increased occurrence of extremely low temperatures, the negative effects on plants, especially on growth and development, from cold stress are becoming more and more serious. In this research, strand-specific RNA sequencing (ssRNA-seq) was used to explore the dynamic changes in the transcriptome landscape of *Arabidopsis thaliana* exposed to cold temperatures (4°C) at different times. In total, 7,623 differentially expressed genes (DEGs) exhibited dynamic temporal changes during the cold treatments. Gene Ontology (GO) and Kyoto Encyclopedia of Genes and Genomes (KEGG) pathway enrichment analysis showed that the DEGs were enriched in cold response, secondary metabolic processes, photosynthesis, glucosinolate biosynthesis, and plant hormone signal transduction pathways. Meanwhile, long non-coding RNAs (lncRNAs) were identified after the assembly of the transcripts, from which 247 differentially expressed lncRNAs (DElncRNAs) and their potential target genes were predicted. 3,621 differentially alternatively spliced (DAS) genes related to RNA splicing and spliceosome were identified, indicating enhanced transcriptome complexity due to the alternative splicing (AS) in the cold. In addition, 739 cold-regulated transcription factors (TFs) belonging to 52 gene families were identified as well. This research analyzed the dynamic changes of the transcriptome landscape in response to cold stress, which reveals more complete transcriptional patterns during short- and long-term cold treatment and provides new insights into functional studies of that how plants are affected by cold stress.

## Introduction

Cold weather is a major environmental stress factor that challenges plant growth, development, yield, and geographical distribution (Jha et al., 2017; Zhang et al., 2019). Many important crops such as rice, maize, soybean, and cotton are considerably affected by exposure to cold stress during their life cycles (Thakur et al., 2010). For example, under cold temperatures, rice yields will decrease by 30-40% (Andaya and Mackill, 2003). When plants were exposed to low temperatures, a series of cellular and molecular responses are activated including transcription, posttranscriptional processing, posttranslational modification, and protein turnover that allows the plants to adapt to cold stress (Thomashow, 2010; Knight and Knight, 2012; Kim et al., 2015; Zhu, 2016). Cold stress is divided into two different types depending on the temperature, namely chilling stress (0–15°C) and freezing stress (< 0°C), which are regulated by different biological mechanisms (Chinnusamy et al., 2007). Chilling stress usually causes adverse effects on membrane fluidity, reactive oxygen species (ROS) homeostasis, photosynthesis, and energy metabolism in plants (Ruelland et al., 2009). Freezing stress results in intercellular ice formation, which causes membrane lesions and structural damage to the cell and causes more serious injuries to the plants (Pearce, 2001). Most plants, including *Arabidopsis*, wheat (*Triticum aestivum* L.), and barley (*Hordeum vulgare* L.), have evolved cold acclimation mechanisms that increase their tolerance to cold stress after exposure to non-freezing temperatures (Chinnusamy et al., 2007).

Cold acclimation involves a series of complex molecular, physiological, and biochemical regulations (Knight and Knight, 2012; Barrero-Gil and Salinas, 2013). In *Arabidopsis*, C-repeat/DERB binding factors (CBFs) are key transcription factors that are critical for activating cold acclimation (Jia et al., 2016; Zhao et al., 2016; Liu et al., 2018). CBF proteins bind CRT/DRE cis-elements, which contain a conserved CCGAC sequence within cold-regulated (*COR*) genes, thereby regulating their expression (Thomashow, 2010; Jia et al., 2016). The *COR* genes encode various proteins, including osmolyte and cryoprotective proteins, that protect plant cells against freezing stress (Chinnusamy et al., 2007). When the plants are exposed to cold stress, *CBF* genes are rapidly induced, and the resulting expressed proteins activate the downstream *COR* genes (Gilmour et al., 1998; Vogel et al., 2005). The expression of *CBFs* is positively and negatively regulated by a set of TFs. For example, INDUCER OF CBF EXPRESSION 1 (ICE1) and CALMODULIN-BINDING TRANSCRIPTION ACTIVATORS (CAMTA) proteins (CAMTA1-5) activate *CBFs* transcription (Chinnusamy et al., 2003; Doherty et al., 2009; Kim et al., 2013; Kidokoro et al., 2017). In contrast, MYB15 and phytochrome-interacting factors (PIFs) function as transcriptional repressors (Agarwal et al., 2006; Leivar et al., 2008). Several hormone signaling pathways have been implicated in the transcription of *CBF*s genes, including ETHYLENE INSENSITIVE 3 (EIN3) and EIN3-BINDING F-BOX proteins (EBF1, EBF2) in the ethylene signaling pathway as well as JASMONATE ZIM-DOMAIN (JAZ) proteins in the jasmonic acid signaling pathway (Shi et al., 2012; Hu et al., 2013; Jiang et al., 2017). Remarkably, the lncRNA *SVALKA* induced the expression of a cryptic antisense *CBF1* lncRNA (*asCBF1*) during cold acclimation, which in turn regulated the expression of *CBF1* (Kindgren et al., 2018). In addition, 135 differentially expressed lncRNAs were identified in the early stage of cold treatment in *Arabidopsis* (Calixto et al., 2019). Thus, the cold response is a complex and multi-level regulated process.

Vernalization is a phenomenon that enables plants to flower in the next growing season after being exposed to long-term cold (Kim et al., 2009). In *Arabidopsis*, FLOWERING LOCUS C (FLC) is a key regulator that acts as a suppressor of flowering during the cold response (Michaels and Amasino, 1999). The *cold induced long antisense intragenic RNA* (*COOLAIR*) and *COLD ASSISTED INTRONIC NON-COLDING RNA* (*COLDAIR*) are both involved in the epigenetic silencing of the *FLC* locus (Swiezewski et al., 2009; Heo and Sung, 2011). Meanwhile, VERNALIZATION 2 (VRN2) and VERNALIZATION INSENSITIVE 3 (VIN3) are involved in the repression of *FLC* expression (Sung and Amasino, 2004; Wood et al., 2006). While short- and long-term cold treatments affect different biological processes, the difference remains to be explored.

Alternative splicing (AS) is the most significant mechanism responsible for the production of multiple protein isoforms from a single gene (Punzo et al., 2020). Recent studies have identified that AS is an important mechanism involved in the regulation of abiotic stress, such as high salinity, drought, and extreme temperatures (Chen and Manley, 2009; Chang et al., 2014; Calixto et al., 2018; Laloum et al., 2018). In *Arabidopsis*, ∼49.4% of all intron-containing genes were alternatively spliced under salt stress (Ding et al., 2014). In the early stage of cold stress, 7,302 DEGs and 2,442 DAS genes were identified, which have different functions during cold treatments (Calixto et al., 2018). Heat stress induces AS, the major type of which involves intron retention (IR) events (Ling et al., 2018). ABA treatment of *Arabidopsis* seedlings led to ∼83.4% of all intron-containing genes being alternatively spliced (Zhu et al., 2017). In plants, several omics approaches have been effective over the past decade for identifying transcriptional and translational changes in response to abiotic stress (Imadi et al., 2015; Janmohammadi et al., 2015; Yoshida et al., 2015). These techniques have been instrumental in enhancing our understanding of molecular and biological processes in response to cold stress. In particular, global gene expression analysis using RNA-Seq and advanced bioinformatics tools can help to explore the key genes during transcription and post-transcription, as well as the key pathways during abiotic stress (Li et al., 2018).

Since low temperature is a key factor of environmental stress, we were interested in investigating the key genes responsible for enabling resistance to cold stress in *Arabidopsis thaliana*. To accomplish this, we employed ssRNA-seq to identify and characterize the DEGs and DAS genes in response to different lengths of cold stress. In addition, the cold-induced AS, lncRNAs, and TFs as well as their potential target genes were identified and analyzed. Our findings lay a foundation for understanding the transcriptional patterns and biological mechanisms involved in response to short- and long-term cold responses.

## Materials and methods

### Plant materials and growth conditions

*Arabidopsis FRI-Col* (Col-0 with a functional *FRI* allele) seeds were surface-sterilized and sown on 0.6% agar plates containing 1/2 Murashige and Skoog (MS) medium. After 3 days of incubation at 4°C, the plates were transferred to a long-day greenhouse (LD, 16 h of light/8 h of dark, 21°C, full-spectrum white fluorescent light with 95 μmol⋅m^–2^⋅s^–1^) for 10 days, after which the seedlings were placed in a short-day plant growing chamber (8 h of light/16 h of dark, full-spectrum white fluorescent light with 30 μmol⋅m^–2^⋅s^–1^) at 4°C for 0 hour (0 h), 6 hours (6 h), 24 hours (24 h), 3 weeks (3 W), and 6 weeks (6 W) to undergo cold treatment. Each experiment underwent three independent replicates for each time point (15 samples in total). The plant tissue samples were rapidly frozen in liquid nitrogen and stored at –80°C.

### Total RNA extraction, library preparation, and sequencing

The total RNA was extracted from the seedlings grown under different cold conditions using an RNA prep Pure Plant Kit (Qiagen according to the manufacturer’s instructions). Purified mRNA was used to construct sequencing libraries with the NEBNext^®^ Ultra™ RNA Library Prep Kit for Illumina^®^ (NEB, United States) following the manufacturer’s recommendations, and index codes were added to attribute sequences to each sample. SsRNA-seq was performed on the Illumina NovaSeq 6000, from which 150 bp paired-end reads were generated. The above work was done in Novogene in Beijing.^1^

### Data processing

Residual adaptor sequences and low-quality bases were removed using the Trimmomatic (v0.36) (Bolger et al., 2014) with a quality score threshold set at 25 and the minimum length of the trimmed read set at 50. The FASTQ files before and after trimming were examined using the FastQC (Wingett and Andrews, 2018). Reference genome files were downloaded from the Ensembl Plants database (TAIR10). The index of the reference genome and clean reads mapping were using the STAR (v2.5.3.a) (Dobin et al., 2013). Reads with a mapping quality score (MAPQ) ≥20 were selected by using the Samtools (v1.15) (Li et al., 2009).

### Identification of long non-coding RNAs

Long non-coding RNAs (LncRNAs) were identified from our transcriptome data using the following procedures. The mapped reads of each sample were assembled and merged in StringTie (v1.3.3b) using a reference-based approach (Pertea et al., 2015). The assembled transcripts were annotated using the GffCompare (Pertea and Pertea, 2020). Subsequently, candidate novel lncRNAs were identified based on the following filtering steps: First, the transcripts with the class codes “u,” “I,” “x,” and “o” were selected. Then, the transcripts with an exon number > 1 and an exon length ≥ 200 nucleotides (nt) were selected. Transcripts with an average FPKM > 0.1 across all samples were then selected. Known ncRNAs (rRNAs, tRNAs, snRNAs, and snoRNAs) were removed by Rfam (Kalvari et al., 2018). Transcripts were predicted coding potential using the Coding Potential Calculator (CPC2) (Kang et al., 2017) and Coding-Non-Coding Index (CNCI) (Sun et al., 2013). The transcripts were then translated into protein sequences using the EMBOSS with 6 frames (Rice et al., 2000), and their coding potential was predicted using HMMER (Mistry et al., 2013). Finally, the coding potential of the transcript sequences was predicted using the nr protein database (NCBI), SwissProt database, and the BLAST + (Camacho et al., 2009).

### Combined analysis of long non-coding RNA and mRNA

Read counts and Fragments Per Kilobase of exon model per Million mapped fragments (FPKM) of genes and isoforms were calculated using the RSME (Li and Dewey, 2011). The hclust and dict functions were used for hierarchical clustering analysis based on the expression value of FPKM in the different samples, and visualization of the data was enabled by the ggtree (Yu et al., 2017). Pearson’s correlation coefficient was calculated and visualized by the ggcor (Huang et al., 2020). The t-distributed stochastic neighbor embedding (t-SNE) and principal component analysis (PCA) were calculated and visualized using the Rtsne, FactoMineR (Lê et al., 2008), and ggplot2, respectively. Analysis of the DEGs between different cold responses was identified if the abs(log2FoldChange) ≥ 1.5 and false discovery rate (FDR)<0.05 using the DESeq2 (Love et al., 2014).

### Alternative splicing events and differentially alternatively spliced genes analysis

We used the IsofromSwitchAnalyzeR to identify AS events and DAS genes (Vitting-Seerup and Sandelin, 2019). AS events were classified into five basic types, including Exon skipping (ES), Mutually exclusive exon and multiple Exon Skipping (MXE), Alternative 5’ splice site (A5), Alternative 3’ splice site (A3), and intron retention (IR). Analysis of the DAS genes between different cold responses was identified if the differences in the isoform fraction (dIF) > 0.1.

### Time-series analysis

Time-series analysis was performed using the Mfuzz (Kumar and Futschik, 2007) based on the FPKM data of the DEGs and DAS genes. The FPKM data were filtered to exclude genes with low standard deviations and then standardized for further analysis.

### Function and pathway enrichment analysis

To identify the biological function of the DEGs and DAS genes identified during the cold treatments. Functional enrichment analyses, including GO^2^ and KEGG pathway^3^ enrichment analyses, were performed using the clusterProfiler (Ashburner et al., 2000; Kanehisa and Goto, 2000; Wu et al., 2021). The GO terms were divided into three separate subgroups: biological process (BP), cellular component (CC), and molecular function (MF). The enriched GO terms and KEGG pathways were identified based on the cut-off criterion of FDR < 0.05.

### Prediction of long non-coding RNAs target genes

The WGCNA was employed to create a co-expression network using the FPKM data of the DEGs and DAS genes screened above (Langfelder and Horvath, 2008). In the WGCNA pipeline, the optimal soft threshold was set at 22, where the fitting curve approached 0.9. Similar modules with a height cut-off value of 0.25 were combined, and the threshold of the number of genes in each module was set to 100. To identify the modules that significantly correlated with cold treatment time, we performed Pearson’s correlation analysis and computed the Student’s asymptotic P-value of each module at all cold treatment time points. The coding genes within 50 kb upstream of the 5’ end or downstream of the 3’ end of each lncRNA sequence were identified as potential cis-targets. Finally, the predicted regulatory networks between the lncRNAs and their potential targeted genes were visualized using Gephi (Bastian et al., 2009).

### Transcription factor annotation and enrichment analysis

The *Arabidopsis* transcription factors (TFs) were downloaded from the plant TF database PlantTFDB.^4^ The functional binding sites of each TF were downloaded from FunTFBS.^5^ The DEGs and DAS genes with TF-binding sites in the promoter regions (–500– + 100) were identified as potential target genes. The predicted regulatory networks between enriched TFs and their potential target genes were visualized using Gephi.

### Quantitative real-time polymerase chain reaction (qRT-PCR) analysis

RNA extraction was performed using the hot phenol method (Yang et al., 2017). Genomic DNA contamination was removed by DNase I (Roche, 04716728001) following the manufacturer’s guidelines. Chloroform extraction and ethanol precipitation were performed to purify the RNA after treatment with the DNase I. From the RNA, cDNA was synthesized using the HiScript II 1st Strand cDNA Synthesis Kit (Vazyme, R211-01) with Oligo dT (15,18 and 21). The cDNA was then diluted 20-fold before undergoing qPCR with the ChamQ SYBR qPCR Master Mix (Vazyme, Q311-02). *AT2G39720* (*RHC2A*) and *AT5G25760* (*UBC21*) were used as the reference genes.

## Results

### Genome-wide identification of long non-coding RNAs

*FRI-Col* is *Col*-0 with an introgressed active *FRIGIDA* allele (*FRI-sf2*) and served as a target to better understand the effects of *Arabidopsis* on cold stress. To investigate the temporal transcriptomic changes in *Arabidopsis FRI-Col* during cold treatment at different time points, we conducted transcriptome analysis of *FRI-Col* seedlings exposed to 4°C temperatures for 0 h, 6 h, 24 h, 3 weeks, and 6 weeks (Supplementary Figure 1). A total of 15 independent libraries were generated and sequenced at a depth of >40 million reads per library (Supplementary Table 1). To identify novel lncRNA transcripts in *Arabidopsis* during the cold treatment, mapped reads were assembled *de novo*, from which a total of 76,905 transcripts corresponding to 31,627 loci were initially generated. After filtering based on the class code, transcript length, exon number, and FPKM, 1,088 transcripts were chosen to predict the protein-coding capacity. After the filtering steps, 114 reliably expressed novel lncRNAs corresponding to 86 loci were obtained (Supplementary Figure 2A and Supplementary Table 2). The novel lncRNA genes and known lncRNA genes were then combined for further analysis (Supplementary Figure 2B).

In addition, the total exon numbers and transcript lengths of the lncRNA genes were compared with the 27,628 coding genes in the TAIR10 genome. The exon number in the lncRNA gene sequences ranged from 1 to 18, and the vast majority (82.4%) of the lncRNA genes contained one exon, the proportion which was much higher than the coding genes. The average number of exons per lncRNA gene was 1.27, which was less than the average (5.41 exons) found in the sequences of the coding genes (Supplementary Figure 2C). The majority of the lncRNA genes had shorter sequences than those of the coding genes. For example, 85.21% of the lncRNA genes ranged in length from 200 to 800 nt, and most were in the range of 200–400 nt. In contrast, 83.53% of the coding genes were longer than 1,000 nt, and most were longer than 2,000 nt (Supplementary Figure 2D). To verify the expression levels of the lncRNA genes, four lncRNA genes were chosen as representative examples, and their expression levels were quantified by qRT-PCR. The qRT-PCR results demonstrated that the expression levels were consistent with the RNA-seq data (Supplementary Figures 2E–H). In total, 86 novel lncRNA genes were identified, and there were significant differences in sequence characteristics between lncRNA genes and coding genes.

### Transcriptome divergence occurs in cold accumulation

**FIGURE 1:**
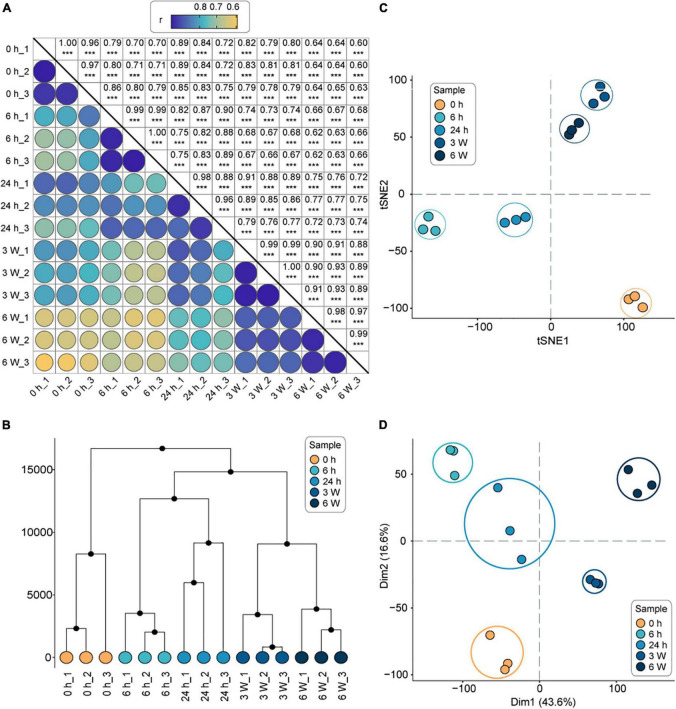
The overall analysis of the transcriptomes of *FRI-Col* in different times of cold treatment. **(A)** Pearson’s correlation coefficient of gene expression values of all samples. ***, *P* < 0.001. **(B)** Hierarchical clustering analysis (HCA) of gene expression values of all samples. **(C)** t-Distributed Stochastic Neighbor Embedding (t-SNE) of gene expression values of all samples. **(D)** Principal component analysis (PCA) of gene expression values of all samples. Axes show principal components (PC) 1, PC2 which explained 60.2% of the variance. Each color represents one time point and three biological replicates were performed for each time point in separate experiments (15 samples in total) in **(A–D)**.

The clean reads were mapped to the *Arabidopsis* genome, resulting in the identification of 24,612 total genes in the 15 samples. Following, the number of expressed genes and their FPKM in each sample were analyzed (Supplementary Table 3). The Pearson correlation coefficients between the three biological replicates were statistically significant (correlation coefficients > 0.9, *P* < 0.001), indicating that the transcriptome data were suitable for the analysis of time point-specific gene expression (Figure 1A). The HCA revealed that the transcriptional profiles of the replicates were consistent at each time point, whereas the profiles at different time points were significantly different (Figure 1B). The t-SNE analysis revealed that the short-term cold treatments (6 h, 24 h) were separate closest to distribution, and long-term cold treatments (3 weeks, 6 weeks) were separate closest to distribution, which were furthest from 0 h (Figure 1C). Finally, the PCA was sufficient to separate the samples into the five groups (Figure 1D), which was consistent with the HCA and t-SNE results. Therefore, the 0 h timepoint clustered away from all other timepoints, indicating a dramatic change in gene expression profile during the cold treatments. The relationship between the short-term cold treatments suggested that 6 h and 24 h timepoints had similar intrinsic gene expression profiles, indicating that short-term cold treatment might have similar biological processes. On the other hand, the long-term cold treatments displayed different gene expression profiles compared to the short cold treatments. Therefore, the genes respond to short- and long-term cold treatments might be involved in different biological processes.

### Temporal gene expression during different cold time points

**FIGURE 2:**
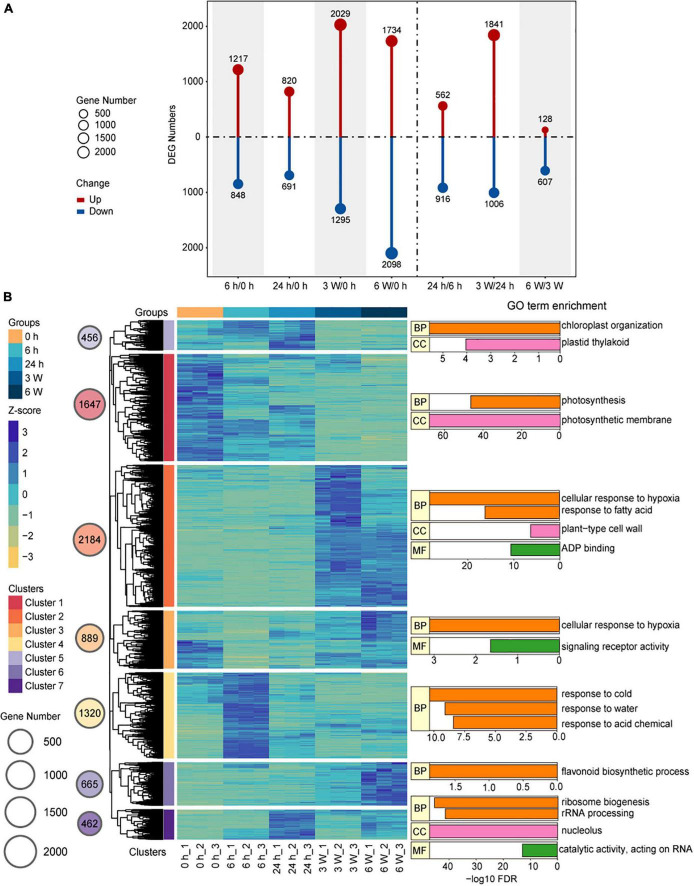
Changes of the transcriptome during cold treatment in *Arabidopsis*. **(A)** Bar graph showing total numbers of differentially upregulated (red) and downregulated (blue) genes at each pairwise time point. **(B)** Hierarchical clustering and heat map of *Arabidopsis* DEGs and Key GO Terms. The groups, Z-score values, and Clusters were assigned different colors. The gene number of the different clusters was shown on circle size. Bar plot of –log10 transformed FDR values are shown. BP, biological process; MF, molecular function; CC, cellular component.

Hierarchical clustering analysis (HCA) provided a global view of the divergence of gene expression profiles during the cold treatment at different time points. DEGs were identified by comparing the samples between different time points, from which a total of 7,623 DEGs were identified (Supplementary Table 4). In the samples that underwent short-term cold treatments, there were 2,065 DEGs for 6 h/0 h, 1,511 DEGs for 24 h/0 h, and 1,478 DEGs for 24 h/6 h. For the long-term cold treatments, there were 3,324 DEGs in 3 weeks/0 h, 3,832 DEGs in 6 weeks/0 h, 2,847 DEGs in 3 weeks/24 h, and 735 DEGs in 6 weeks/3 weeks (Figure 2A). To verify the expression levels of the DEGs in the samples exposed to both short- and long-term cold treatments, the expression levels of DEGs were quantified by qRT-PCR. The key cold-response genes *CBF3*, *RD29A*, and *COR6.6* were induced by short-term cold treatments, then decreased in the long-term cold treatments (Supplementary Figures 3B–D), the expression patterns of these genes were consistent with the Col-0 (Shi et al., 2012). Whereas, *CBF1* and *CBF2* displayed distinct expression patterns compared to Col-0. These two genes were highly expressed before cold, and the short cold induced expression was disrupted in *FRI-Col*, suggesting a certain level of difference on short-term cold response between *FRI-Col* and Col-0 (Supplementary Figures 3A,G). We also measured the expression levels of the well characterized long-term cold induced gene *VIN3* and repressed gene *FLC* (Kim and Sung, 2013). *VIN3* was induced in 3 W and 6 W samples and *FLC* was repressed by 3 W and 6 W cold treatments, the expression changed of which was not observed in 6 h and 24 h cold treatment samples (Supplementary Figures 4C,I). We also observed continues induction of *KIN1* expression (Supplementary Figure 4B). A few more genes, which displayed changed expression in cold, were also measured by qRT-PCR. All the results displayed similar expression trends between qRT-PCR and the RNA-seq data (Supplementary Figures 3, 4). Together, we found that both short-term cold treatments (6 h/0 h) and long-term cold treatments (3 weeks/0 h, 6 weeks/0 h, 3 weeks/24 h) induced significant changes at the transcription level, indicating different transcription patterns between the short- and long-term cold treatments.

We used the FPKM to analyze gene expression and perform HCA on the 7,623 DEGs, and the results were visualized by Pheatmap. A total of seven co-expressed clusters were generated (Figure 2B and Supplementary Table 5). The gene expression profiles in each cluster showed similar expression patterns. Particularly, Cluster 1 (*n* = 1,647), which represented mostly downregulated genes at all cold treatment stages, was enriched for GO terms related to photosynthesis and photosynthesis membrane, indicating photosynthesis was largely reduced by cold treatment. The clusters with genes upregulated in response to short-term cold treatments, such as Cluster 4 (*n* = 1,320), Cluster 5 (*n* = 456), and Cluster 7 (*n* = 462), were enriched for GO terms associated with response to cold, chloroplast organization, ribosome biogenesis, and rRNA processing. These genes, such as *CBF3* and *COR6.5* in Cluster 4, the FPKM peaked at 6 h and 24 h during the cold treatment, after which they decreased, indicating that these genes responded to the cold stress very quickly and may activate cold tolerance pathways. In contrast, the clusters with genes upregulated in response to long-term cold treatments, such as Cluster 2 (*n* = 2,184), Cluster 3 (*n* = 889), and Cluster 6 (*n* = 665), were enriched for GO terms associated with cellular response to hypoxia, fatty acid, and flavonoid biosynthetic process (Figure 2B and Supplementary Table 6). During long-term cold treatments, the genes related to redox homeostasis were upregulated, causing the levels of intracellular reactive oxygen species (ROS) to rise (Moon et al., 2015). The heat shock proteins (HSPs) are the key regulators that plants used to cope with environment stress such as high temperature, cold, drought, salinity, osmotic, and oxidative stresses (Vierling, 1991; Grigorova et al., 2011; Cao et al., 2012; Li Z. Y. et al., 2016). We also found 31 HSP genes were differentially expressed under long-term cold treatments (Supplementary Figure 5). For example, *HSP17.4*, which links biotic and abiotic stress response with ROS signaling pathway (Sewelam et al., 2019), was up-regulated by long-term cold treatments. These results displayed that the expression patterns of DEGs were distinct and involved in different biological functions depending on the duration of cold stress.

### Different durations of cold stress affect the transcription of differentially alternatively spliced genes

**FIGURE 3:**
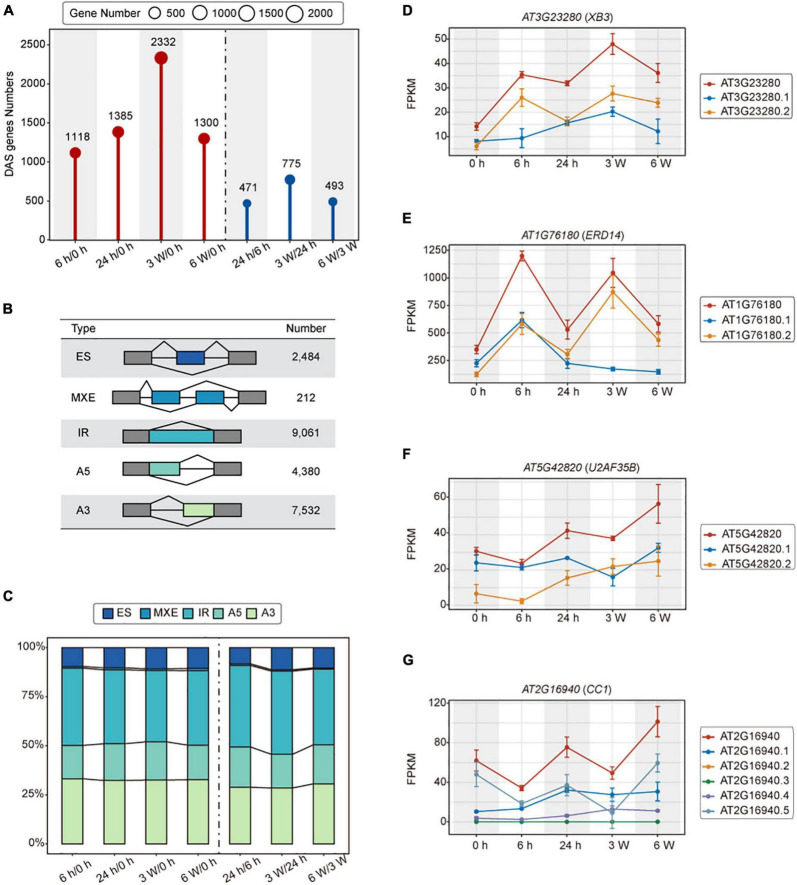
Alternative splicing of the transcriptomes of *FRI-Col* in different times of cold treatment. **(A)** Identified differentially alternatively spliced (DAS) genes in the *Arabidopsis* transcriptome during cold treatment. **(B)** Summary table of AS events detected by the IsofromSwitchAnalyzeR. Exon skipping (ES), Mutually exclusive exon and multiple Exon Skipping (MXE), Alternative 5’ splice site (A5), Alternative 3’ splice site (A3), and intron retention (IR). **(C)** Stacked bar charts showing the AS events percentage at each pairwise time point. Line charts showing the ES, IR, A5, and A3 events in **(D–G)** respectively.

Alternative splicing (AS) is actively involved in various stress responses in plants (Laloum et al., 2018). We identified a total of 3,621 DAS genes in cold treated samples (Figure 3A and Supplementary Table 7). During the short-term cold treatments, 1,118 and 1,385 DAS genes were identified in 6 h/0 h and 24 h/0 h, respectively. Among them, 471 DAS genes were detected in 24 h/6 h. For the long-term cold treatments, there were 2,332 and 1,300 DAS genes identified in 3 weeks/0 h and 6 weeks/0 h, respectively. 775 DAS genes in 3 weeks/24 h and 493 DAS genes in 6 weeks/3 weeks were identified as well (Figure 3A).

To investigate the influence of cold stress on AS, we analyzed the cold treatment transcriptome data using the IsofromSwitchAnalyzeR. A total of 23,669 AS events were grouped to 5 different types. The IR accounted for 9,061 (38.28%) of the total AS events, representing the major type of AS events, followed by A3, A5, ES, and MEE, which accounted for 7,532 (31.82%), 4,380 (18.51%), 2,484 (10.49%), 212 (0.90%) events, respectively (Figure 3B and Supplementary Tables 8, 9). Furthermore, we observed the same AS event trends described above at each time point during the cold stress experiments (Figure 3C). These results suggested that AS contributed to a specificity response during different times of cold treatment, with IR being the major splicing pattern.

Alternative splicing (AS) generates multiple transcripts of a single gene. These transcripts can be translated into different proteins that may perform different biological functions. XB3 is a player in ethylene signaling, which promotes the degradation of ACD11 to attenuate the ABA and salt stress (Li et al., 2020). In the ES events, *XB3* produced transcript isoforms that have different expression patterns in 24 h, but the expression of *XB3* was the highest at 3 weeks (Figure 3D). In IR events, *ERD14* encodes a dehydrin protein whose expression is induced early on in response to dehydration stress. During IR events, the *ERD14* gene produced transcript isoforms that had the highest expression at 6 h and 3 weeks (Figure 3E). *ERD14* is implicated in the protection and activation of redox enzymes, which help to protect plants from oxidative stress (Nguyen et al., 2020). In A5 events, different transcripts of *U2AF35B* showed different expression levels at 3 weeks (Figure 3F). U2AF35B, as a splicing factor, has been found to shuttle between nuclei and the cytoplasm (Park et al., 2017). U2AF35B also interacts with U2AF65b, which is involved in ABA-mediated flowering by regulating the pre-mRNA splicing of *ABI5* and *FLC* (Xiong et al., 2019). In A3 events, *CC1*, also a splicing factor, whose transcript expression levels varied between different time points (Figure 3G). CC1 is involved in the dynamic regulation of Pep-induced immunity through post-translational modifications (Dressano et al., 2020). Based on these data, we demonstrated that cold-dependent AS is an important regulatory mechanism that controls the abundance of cold response genes and transcripts.

### Differences in the enrichment between differentially expressed genes and differentially alternatively spliced genes

The DEGs and DAS gene data sets were largely different, and only 1,045 genes overlapped between the two (Supplementary Figure 6A). For the DEGs, the most significantly enriched GO terms were related to secondary metabolic process, cellular response to oxygen levels, response to cold, photosynthesis, and response to an acid chemical in BP. Thylakoid membrane, photosynthetic membrane, plant-type cell wall in CC. Oxidoreductase active, monooxygenase activity in MF. The most significantly enriched KEGG Pathway was related to photosynthesis and glucosinolate biosynthesis (Supplementary Figure 6B and Supplementary Table 10). These results indicated that the underlying biological functions of DEGs were mostly related to cold response.

For the DAS genes, the most enriched functional terms were related to mRNA metabolic process, mRNA processing, RNA splicing, and regulation of response to stress in BP. Nucleoplasm, nuclear body in CC. Cytoskeletal protein binding, motor active, and ATP binding in MF. The most significant enriched KEGG Pathway was related to spliceosome (Supplementary Figure 6B and Supplementary Table 11). These results indicated that the underlying biological functions of DAS genes were mostly related to the cold response through mRNA-related functions and RNA splicing genes, especially those coding splicing regulators that could be actively alternatively spliced during cold treatment.

Transcriptome analysis of the cold treatment of *Arabidopsis* has been reported previously (Vogel et al., 2005; Carvallo et al., 2011; Leviatan et al., 2013; Gehan et al., 2015; Jia et al., 2016; Zhao et al., 2016). There was considerable overlap between the cold response genes in previous studies and the cold response genes identified in this study (Supplementary Figure 6C and Supplementary Table 12). However, we identified an additional 2,826 DEGs, 2,124 DAS genes, and 212 DElncRNAs in across all the cold-treated samples. We further identified additional 222 DEGs and DAS genes, and 4 DElncRNAs during the short-term cold treatments (Supplementary Figure 6D) and additional 280 DEGs and DAS genes, and 2 DElncRNAs during the long-term cold treatments (Supplementary Figure 6E). These results indicated that different DEGs were detected at different time points during the cold treatment, suggesting that *Arabidopsis* has distinct physiological responses to short- and long-term cold treatments.

### Dynamic changes in the transcriptome of *Arabidopsis* in response to cold treatment

Cold treatment induces ongoing dynamic changes in the transcriptome, resulting in different transcriptional profiles in the plants exposed to short- and long-term cold treatment. We identified 15 distinct genome-wide clusters, each of which contained a unique gene set ranging from 476 to 926 members (Supplementary Figure 7 and Supplementary Table 13). These distinct patterns suggested a precise temporal transcriptional regulation corresponding to the different times of cold exposure.

**FIGURE 4:**
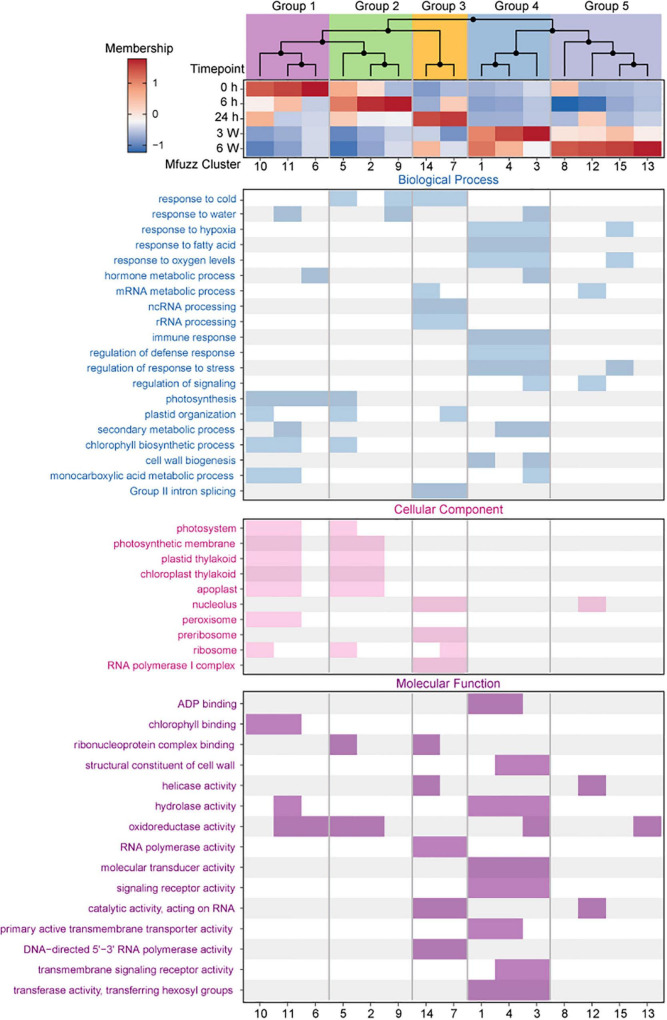
Expression dynamics reveal a temporal ordering of biological processes during cold treatment. **(Top)** Hierarchical clustering and heat map of Mfuzz cluster core expression profiles. **(Bottom)** GO enrichment analysis with clusterProfiler.

To further analyze the dynamic changes in gene expression profiles at different time points, the core genes of 15 clusters were selected based on the membership value α > 0.65. The membership value of the core genes was also used for HCA. Hierarchical clustering of the core expression profiles from 15 identified Mfuzz clusters resulted in five temporally ordered groups that showed progressive expression dynamics as a cold accumulation process (Figure 4). A time-dependent shift in GO terms was observed between the five ordered groups, reflecting the different potential biological functions in the various stages of the cold response (Supplementary Table 14).

Group 1 (Cluster 6, 10, 11) comprised early responsive genes to cold stress that had high expression levels at 0 h but were downregulated at different cold treatment time points thereafter (Figure 4 and Supplementary Figure 7). GO enrichment analysis identified that these genes were related to photosynthesis, photosystem, and chlorophyll binding (Figure 4), which revealed that plant photosynthesis-related biological processes were continually being inhibited throughout the cold treatment. The genes in Group 2 (Cluster 2, 5, 9) exhibited highest expression levels at 6 h (Figure 4 and Supplementary Figure 7). The GO terms enriched in this group were related to response to cold and oxidoreductase activity (Figure 4), indicating that these genes were likely the first batch of genes responded to the cold stress. The genes in Group 3 (Cluster 7,14) exhibited relatively constant upregulation at 24 h, and the GO terms enriched in this group were related to RNA processing, ribosome, and RNA polymerase activity (Figure 4 and Supplementary Figure 7). The genes in Group 4 (Cluster 1, 3, 4) and Group 5 (Cluster 8, 12, 13, 15) exhibited constant upregulation during the long-term cold treatments (Figure 4 and Supplementary Figure 7) and enriched in these groups were related to hypoxia, fatty acid, oxygen levels, regulation of response to stress, and signaling receptor activity (Figure 4 and Supplementary Figure 7). These data suggest that functional distinct groups of genes are required for different length of cold treatments.

### Regulatory functions of long non-coding RNAs in *Arabidopsis*

**FIGURE 5:**
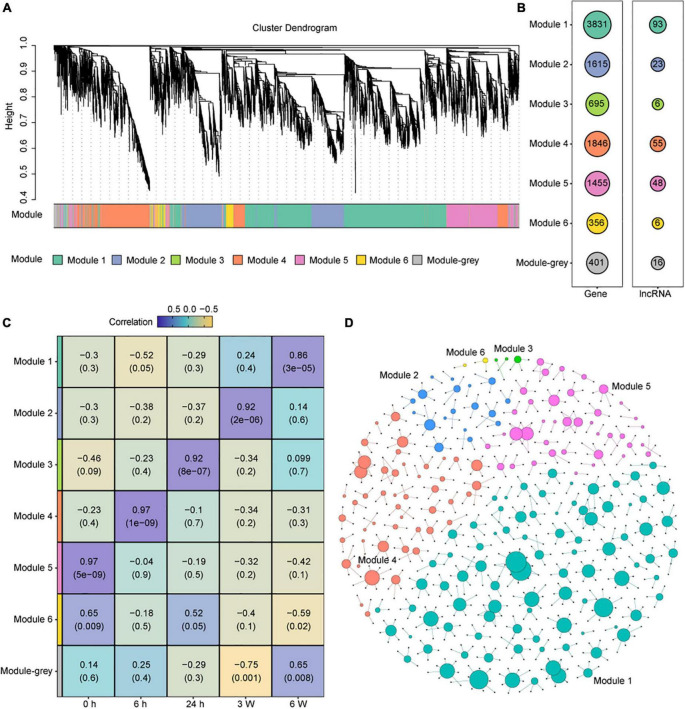
Construction and identification of the co-expression modules associated with the timepoints, based on the FPKM values of DEG and DAS genes. **(A)** WGCNA cluster dendrogram showing co-expression modules identified in 15 samples of 5 timepoints. Modules are assigned by distinct colors, respectively. **(B)** The number of DEGs and DElncRNAs in different Modules. **(C)** Correlations between modules and cold treatment timepoints. The correlation is estimated by the Pearson correlation coefficient method. **(D)** Predicted regulatory networks based on DElncRNAs and their potential targeted genes in DEG and DAS genes. Different colors represent different modules and the big dots represent lncRNAs. The detailed information was showing in Supplementary Table 16.

As a set of lncRNA were identified, we tried to elucidate the potential biological functions of lncRNAs involved in cold stress. We identified co-expression modules from the transcriptomic data. In total, seven co-expression modules were obtained, and seven colors were assigned for visual distinction (Figure 5A). All non-co-expressed genes were gathered into the gray module (Figure 5A and Supplementary Table 15). Each of the modules contained a unique gene set that ranged from 356 to 3831 members, and the lncRNAs set ranged from 6 to 93 (Figure 5B). To assess the correlation between each module and the cold treatment time points, a correlation analysis between the module and cold treatment time point was performed. We found that Modules 5, 4, 3, 2, and 1 were significantly and positively correlated with the 0 h, 6 h, 24 h, 3 W, and 6 W cold treatment, respectively (correlation > 0.85, P-value < 0.001) (Figure 5C). These results indicated that the modules exhibited time-specific expression.

Recent evidence supports that some lncRNA loci act locally to regulate the expression of nearby coding genes (Engreitz et al., 2016). Thus, we selected 50 kb upstream and downstream of the DElncRNAs to predict the potential target mRNAs, and the lncRNA-mRNA networks were created by Gephi (Figure 5D and Supplementary Table 16). We found 199 DElncRNAs and their 571 potential target genes in this network. In Module 5, which was significantly correlated with 0 h, 39 of the 48 DElncRNAs were predicted to target 79 potential mRNAs, including photosynthesis-related genes and Cytochrome P450 family genes. The lncRNA *AT2G08660* was predicted to regulate the expression of two P450 genes *CYP710A1* and *CYP710A2* that are involved in stress response (Morikawa et al., 2006). *AT2G08660* displayed obvious positive correlation with *CYP710A1* and *CYP710A2* measured by qRT-PCR, which were consistent with the RNA-seq data (Supplementary Figures 8A–C). In Module 4, which was significantly correlated with 6 h, 47 of the 55 DElncRNAs potentially target 111 genes. LncRNA *AT2G09195* was predicted to positively regulated *CYSA* that encodes a protein with cysteine proteinase inhibitor activity and increasing the salt, drought, oxidation, and cold tolerance (Zhang et al., 2008). The expression levels of them showed positive correlation similar as RNA-seq data (Supplementary Figures 8E–F). *MSTRG.17059* as a novel lncRNA was predicted to positively regulated *GA20OX1*, which encodes gibberellin 20-oxidase and response to abiotic stresses, such as cold and drought, by suppressing the biosynthesis of GA in *Arabidopsis* (Chen H. I. et al., 2019). In Module 3, which was significantly correlated with 24 h, 3 of the 6 DElncRNAs and 5 potential target genes were predicted. In Module 2, which was significantly correlated with 3 weeks, 18 of the 23 DElncRNAs and 42 potential target genes were predicted. In Module 1, which was significantly correlated with 6 weeks, 89 of the 93 DElncRNAs and 331 potential target genes were predicted. The lncRNA *AT5G09175* was predicted to negative regulated *AT5G64110*, which encodes a protein of the Peroxidase superfamily that is known to respond to oxidative stress (Dong et al., 2006). *MSTRG.21822* as a novel lncRNA was predicted to positively regulated *MAP18*, which encodes a protein with seven repeated VEEKK motifs and plays an important role in chilling tolerance and ABA response by activating CBF- and SnRK2-mediated transcriptional regulatory network (Wang et al., 2018). We also performed qRT-PCR to validated some of these predictions, such as *MSTRG. 17059* regulating *GA20OX1* (Supplementary Figures 8G–H), *AT5G09175* targeting *AT5G64110* (Supplementary Figures 8J–K), *MSTRG.21822* regulating *MAP18* (Supplementary Figures 2E, 8L). We also measured the expression levels of several genes that predicted to be regulated by lncRNAs. The qRT-PCR data were consistent with the RNA-seq data (Supplementary Figures 8D,I). The above results indicated that lncRNAs may regulate different potential target genes, which contributes cold adaption.

### Identification and verification of transcription factors and their potential targeted genes

**FIGURE 6:**
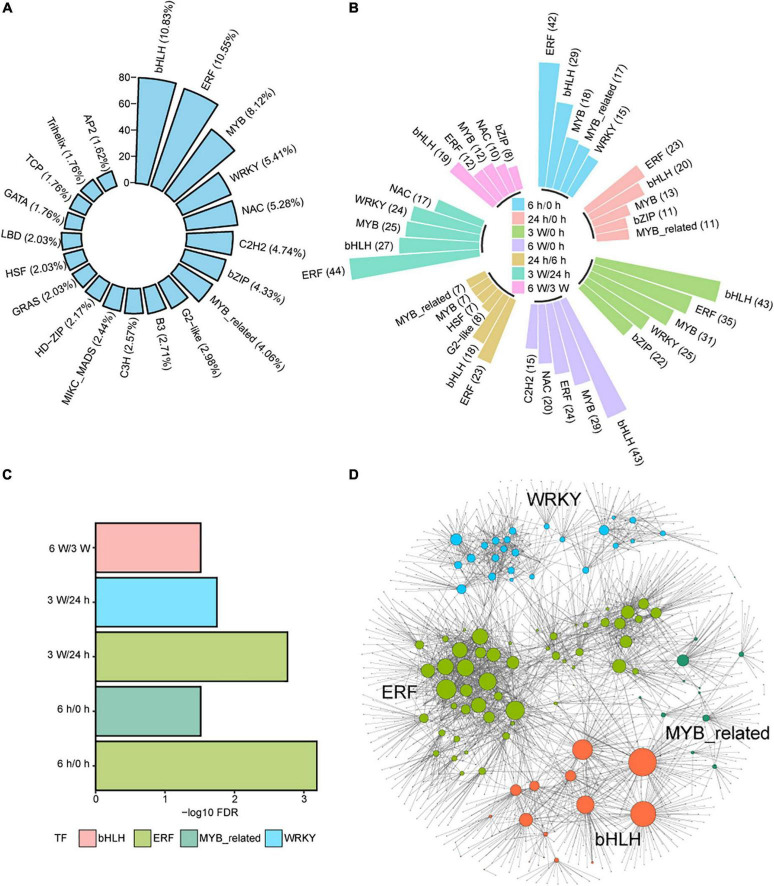
The regulatory networks of differently expressed TFs during cold treatment. **(A)** Bar plot showing the distribution of the top 20 differently expressed TFs in total DEG and DAS genes. **(B)** Bar plot showing the distribution of the top 5 of differently expressed TFs in DEG and DAS genes at each time point. **(C)** Enrichment analysis of TF DNA binding motifs within the promoter regions of DEG and DASs genes at each time point. **(D)** Predicted regulatory networks based on enriched TFs and their potential targeted genes in DEG and DAS genes. Different colors represent different TFs and the big dots represent TFs. The detailed information was showing in Supplementary Table 18.

Transcription factors (TFs) are important regulators that participate in the response to cold stresses. Among 1717 TF coding genes, 739 were expressed in our transcriptome data set. These TFs belonged to 52 TF families, such as bHLH (10.83%), ERF (10.55%), MYB (8.12%), WRKY (5.41%), NAC (5.28%), C2H2 (4.74%), bZIP (4.33%), and MYB_related (4.06%) (Figure 6A and Supplementary Table 17). Some of these TFs were distributed in DEGs and DAS at different time points (Figure 6B), indicating that TFs play important roles in short- and long-term cold treatments.

Next, enrichment analysis for the DEG and DAS TFs was performed. The ERF and MYB_related TFs were significantly enriched at 6 h/0 h, while The ERF and WRKY TFs were significantly enriched at 3 weeks/24 h (Figure 6C). The bHLH TFs were significantly enriched at 6 weeks/3 weeks (Figure 6C). The results indicated that TFs play different functions on the duration of cold treatment. However, the ERF TFs were significantly enriched in both short- and long-term cold treatments, indicating this family of TFs likely play an important regulatory role in both short- and long-cold.

To investigate the links between TFs and other DEGs and DAS genes in cold response, we used the functional binding sites of enriched TFs and the promoter sequence of DEGs and DAS genes to predict target genes (Supplementary Table 18), from which we identified 109 TFs and 1305 potential target genes (Figure 6D). MYB-related TFs may regulate target genes associated with photosynthesis. For example, *MYBD* expression increased in response to light or cytokinin, and MYBD enhanced anthocyanin biosynthesis via repression of MYBL2 (Nguyen et al., 2015). MYBD was predicted positively regulated the photosystem-related gene *PSBTN* (Chen Y. E. et al., 2019). The decreased expression of *PSBTN* was detected along the repression of *MYBD* in the cold measured by qRT-PCR and RNA-seq (Supplementary Figures 9A,B). AIB is a bHLH TF that was controlled by MYC2, which is the key factors to control JA catabolism (Zhang et al., 2020). AIB was predicted to repress regulate the photosynthetic electron transport chain genes *PETC* at 3 weeks cold (Zoulias et al., 2021). Negative correlation of expression levels was measured *PETC* (Supplementary Figures 9C,D). However, this anti-correlation was disrupted at other time points, indicating complicated interactions between AIB and *PETC* (Supplementary Figures 9C,D). We also measured the expression levels of several genes that predicted to be regulated by TFs. The qRT-PCR data were consistent with the RNA-seq data (Supplementary Figures 9E–I), displaying that RNA-seq data were based prediction should be wright at the gene expression level. However, further research on the biological roles of these TFs and their potential target genes in the cold response is warranted.

## Discussion

### Short- and long-term cold treatments have different effects on transcriptome of *Arabidopsis*

In *Arabidopsis*, dynamic changes in transcriptome levels and molecular mechanisms underlying short- and long-term cold treatments remain to be explored. Previous studies identified gene expression patterns and alternative splicing events specifically in short-term cold treatments (Calixto et al., 2018) as well as DEGs in vernalization (Li et al., 2021). These prior reports provided cold stress-specific information to address the changes in transcriptome levels during cold stress. However, the connections among DAS, DEG, and lncRNA were not addressed. Therefore, a more complete transcriptome analysis of *Arabidopsis* during the exposure to cold temperatures will help to elucidate these dynamics changes, identify the molecular mechanisms involved in adaptation to cold temperatures, and facilitate the identification of important regulatory factors involved in these mechanisms.

We performed a detailed transcriptomic analysis of *Arabidopsis* at 0 h, 6 h, 24 h, 3 weeks, and 6 weeks during exposure to cold temperatures (4°C) by ssRNA-seq. We identified several thousand DEGs, suggesting that the expression of genes in *Arabidopsis* changed dynamically during the cold exposure (Figure 2). A set of genes involved in photosynthesis and photosystem were downregulated during the cold treatments, indicating that the photosynthesis is repressed in response to cold stress, which corroborated previous studies (Xi et al., 2020). The cold stress caused down-regulation of light absorption and inhibited the photosynthetic efficiency of plants could protects plants from photo-oxidative damage (Oquist and Huner, 2003). Another study found that long-term, stress-induced stomatal closure and damage to the photosystems also resulted in a decrease in photosynthetic efficiency (Yamori et al., 2014). In our study, after 6 h cold, the expression of genes responding to cold, such as *CBF3* genes and *COR414*, was up-regulated (Supplementary Figures 3B, 9I), indicating a quick response of these genes during cold treatment. After 24 h, the genes involved in ribosome biogenesis and rRNA processing were upregulated (Figure 2B). Ribosome biogenesis is one of the most energy-demanding processes in the cell and is usually associated with cellular stress (Grummt, 2013). Ding and colleagues reported that the expression level of *CBF1*, *CBF2*, and *CBF3* were significantly increase at 4°C for 3 h. The COR genes including *COR15A* and *KIN1* were significantly increase at 4°C for 24 h (Ding et al., 2018). These results were similar with transcriptome data and suggest that *CBF* and *COR* genes play an important role in short-term cold treatment. Moreover, we found that there was a significant difference in the transcription level between short-term (6 h, 24 h) and long-term (3 weeks, 6 weeks) cold treatments based on correlation analysis and HCA (Figure 1). Long-term cold treatments were found to induce the expression of *VIN3* (Supplementary Figure 4C), which enabled vernalization response (Bond et al., 2009; Kim and Sung, 2017). Long-term cold treatments also disrupt redox homeostasis in the cell, which leads to the accumulation of ROS (Moon et al., 2015). Meanwhile, the 31 differentially expressed *HSP* genes were identified, which are associated with the ROS and play a key role in long-term cold treatments (Sewelam et al., 2019). Based on these results, gene transcription in *Arabidopsis thaliana* was heterogeneous under short-term and long-term cold treatments.

### Long non-coding RNAs regulate plant tolerance to cold stress

Previous studies have reported that lncRNAs play an important role in response to abiotic stress by directly or indirectly acting on functional genes in plants (Yu et al., 2019). In *Arabidopsis*, lncRNAs have been identified under various abiotic stresses, including cold, heat, salt, drought, and high light stress, using bioinformatics approaches (Di et al., 2014). *DROUGHT INDUCED lncRNA* (*DRIR*), a novel positive regulator responding to drought and salt stresses, is expressed at low level under normal conditions but can be significantly activated by drought, salt stress, and ABA exposure (Qin et al., 2017). *SVALKA*, another lncRNA, is transcribed from the antisense strand between *CBF3* and *CBF1* genes, which regulates cold tolerance (Kindgren et al., 2018). *TE-lincRNA11195* is an abiotic stress-induced TE-lincRNA whose expression levels were upregulated after exposure to high salt, ABA, and cold temperatures (Wang et al., 2017).

Long non-coding RNAs (lncRNAs) also regulate plant growth and development. The expression of some lncRNAs is regulated by environmental conditions. *CDF5 LONG NON-CODING RNA* (*FLORE*) lncRNA that is transcribed into the antisense orientation relative to *CYCLING DOF FACTOR 5* (*CDF5*), which encodes a circadian protein; these two genes are involved in managing circadian rhythms (Henriques et al., 2017). *Alternative Splicing Competitor* (*ASCO*) lncRNA hijacks nuclear speckle RNA-binding protein (NSR) by competitively binding to mRNAs that modulate the AS of RNAs to regulate lateral root development (Bardou et al., 2014). The key gene in vernalization and autonomous pathways is *FLC*, which is a negative regulator of flowering (Yang et al., 2017). *COOLAIR* negatively regulates *FLC*, which is downregulated during exposure to cold temperatures, and its downregulation is maintained thereafter, allowing the ability to flower in the warmer periods following (Swiezewski et al., 2009).

The transcriptome data obtained in this study identified 76,905 transcripts corresponding to 31,627 loci (Supplementary Figure 2A). After basic filtering, the potential coding capability was predicted, from which 114 novel lncRNAs on 86 loci were identified (Supplementary Figure 2B). Based on differential expression analysis and different AS analyses, a total of 918 lncRNAs were identified, including 218 DElncRNAs and 54 DAS lncRNAs (Supplementary Figure 6A). Based on WGCNA, seven co-expression modules were obtained (Figure 5A), five of which were highly correlated with five time points, indicating that the expression of DElncRNAs changed dynamically during cold (Figure 5C). In addition, their potential target genes were predicted which included genes in photosynthesis, cytochrome P450s, cold response and so on (Figure 5D and Supplementary Table 15). The analysis revealed that the DElncRNAs have key biological functions in the cold response. These results led to the identification of candidate lncRNAs and potential interacting coding genes in response to cold stress of varying durations. We found tight linkage between lncRNA and target genes (Supplementary Table 15). It will be important to address the function of these lncRNAs further in cold response.

### Cold response induces alternative splicing

Alternative splicing (AS) is an important regulatory mechanism on gene expression that can regulate mRNA level and increase proteome diversity (Laloum et al., 2018). In plants, AS is markedly affected by environmental stresses, which impact plant growth and development. Current research suggests that more than 61% of intron-containing genes undergo AS in *Arabidopsis* (Marquez et al., 2012). Transcriptome sequencing has confirmed this phenomenon. For example, abiotic stress was found to significantly modify AS events in plants (Filichkin et al., 2010; Marquez et al., 2012). Salt stress induced the AS events at more than 6,000 genes by promoting the usage of non-canonical splice sites in *Arabidopsis* (Feng et al., 2015). Extreme temperatures (42–45°C) also led to the formation of a new splice variant *HsfA2-III* of *HsfA2*, which uses a splice site in the intron of *HsfA2* (Liu et al., 2013). In one study, 2,442 DAS genes were identified after short-term cold treatments, indicating AS is also sensitive to cold temperature (Calixto et al., 2018).

In this study, a total of 3,621 DAS genes were identified (Figure 4A). Of them, 53 DAS genes were associated with the spliceosome, and 72 DAS genes were associated with RNA splicing in response to cold temperatures (Supplementary Figure 6B). Therefore, short- and long-term cold stresses induced AS by regulating the expression of genes involved in RNA splicing and spliceosome. Plants regulate the abundance of functional proteins through AS. Although some proteins are most likely unnecessary under normal environmental conditions, plants usually require other proteins to respond when the environment changes. In this case, the expression of genes associated with RNA processing, splicing, and the spliceosome are differentially expressed in AS events. Therefore, cold stress not only regulated gene expression at the transcriptional level but also at the post-transcriptional level. In addition to AS, post-transcriptional regulations may also include protein folding and protein modification.

### The regulator mechanisms of cold response is conserved

To compare the regulatory mechanisms involved in the response to cold stress, we compared our results with previous studies in rice (Shen et al., 2014), *Zea mays* (Waititu et al., 2021), and *Triticum aestivum* L. (Jiang et al., 2022). In these species, photosynthesis was significantly downregulated, while response to cold, secondary metabolic process and cellular response to oxygen levels were significantly upregulated in BP (Supplementary Figure 6B). Meanwhile, the photosynthetic membrane and photosystem were significantly down-regulated in CC, and oxidoreductase activity and signaling receptor activity were enriched in MF (Supplementary Figure 6B). Furthermore, photosynthesis-related pathways were enriched in KEGG. These results indicated that cold treatment affected the same GO and KEGG pathways in different species.

The expression of many TFs was regulated in response to the cold stress. In our study, a total of 739 differentially expressed TFs throughout 52 TF families were identified (Supplementary Table 17). The top 10 TF families included bHLH (10.83%), ERF (10.55%), MYB (8.12%), WRKY (5.41%), NAC (5.28%), C2H2 (4.74%), bZIP (4.33%), MYB_related (4.06%), G2-like (2.98%), and B3 (2.71%). These families were consistent with the TF genes observed in rice (Shen et al., 2014; Kong et al., 2020), *Zea mays* (Lu et al., 2017), *Triticum aestivum* L. (Jiang et al., 2022), durum wheat (Díaz et al., 2019), and *Camellia japonica* (Li Q. et al., 2016) in response to cold temperatures, indicating that these TF families may have similar biological functions and regulatory roles in different plants under cold stress. Meanwhile, bHLH, ERF, and MYB responded to all cold response times, indicating that these TF families might be involved in similar regulatory mechanisms in short-term and long-term cold response.

In summary, under short- and long-term cold treatment conditions, lncRNA and mRNA were expressed at different levels and underwent different AS events, which together promoted the transcriptional reprogramming of *Arabidopsis thaliana* and manifested common phenotypes to resist cold stress (Supplementary Figure 10).

## Data availability statement

The data is avaliable now in the SRA database: https://www.ncbi.nlm.nih.gov/bioproject/PRJNA854395/.

## Author contributions

YL and HY designed the research and wrote the manuscript. YL, YC, XZ, NS, and JZ performed the research. YL and YZL performed the bioinformatics analysis. All authors read and approved the final manuscript.

## Footnotes

1
https://en.novogene.com

2
http://geneontology.org/

3
https://www.genome.jp/kegg/

4
http://planttfdb.cbi.pku.edu.cn

5
http://plantregmap.gao-lab.org/funtfbs_manual.php

## Supplementary material

Supplementary Figure 1 Sample processing overview. **(A)** Time points of the sample are marked by the different labels, including 0 hour (0 h), 6 hours (6 h), 24 hours (24 h), 3 weeks (3 W), and 6 weeks (6 W). **(B)** Phenotypes of *FRI-Col0* at 4°C for 0 h, 3 weeks (3 W), and 6 weeks (6 W). Bars = 1cm.

Supplementary Figure 2 Genome-wide identification of lncRNAs in *Arabidopsis thaliana*. **(A)** Pipeline for identification of lncRNAs. **(B)** Venn diagram showing the number of novel lncRNA genes and known lncRNA genes. **(C)** Distribution of exon numbers of lncRNA genes and coding genes. **(D)** Distribution of sequence length of lncRNA genes and coding genes. **(E–H)** Quantitative real-time PCR (qRT-PCR) validation of lncRNAs in different cold treatments. FPKM values (left) and relative expression (right) of lncRNAs in different cold times.

Supplementary Figure 3 Quantitative real-time PCR (qRT-PCR) validation the DEGs of short-term cold treatments of ssRNA-seq. FPKM values (left) and relative expression (right) of DEGs in different cold times.

Supplementary Figure 4 Quantitative real-time PCR (qRT-PCR) validation the DEGs of long-term cold treatments of ssRNA-seq. FPKM values (left) and relative expression (right) of DEGs in different cold times.

Supplementary Figure 5 Transcriptional profile of differentially expressed the heat shock proteins (HSP) genes in different cold treatment. The data are represented as a heatmap generated with scale = row dependent on FPKM. The color scale is shown on the right.

Supplementary Figure 6 DEG and DAS Analysis of *Arabidopsis* transcriptome during cold treatment. **(A)** Flow chart showing the distribution of the DEGs and DAS genes. **(B)** Most significantly enriched GO terms for DEGs and DAS genes. Bar plot of –log10 transformed FDR values are shown. **(C)** Venn diagram showing the DEGs and DAS genes identified and compared with known cold response DEGs and lncRNAs. **(D)** Venn diagram showing the DEGs and DAS genes in short-term cold treatment and compared with lncRNAs. **(E)** Venn diagram showing the DEG and DAS genes in long-term cold treatments and compared with lncRNAs.

Supplementary Figure 7 Temporal expression dynamics were analyzed by standard-normal FPKM using the Mfuzz. Expression dynamic profiles were labeled 1-15. Each plot shows the profile for each cluster with high membership in warm colors (yellow, red), and low membership values in cool colors (green, blue).

Supplementary Figure 8 Quantitative real-time PCR (qRT-PCR) validation the potential target genes of lncRNAs in different cold treatments. FPKM values (left) and relative expression (right) of DEGs in different cold times.

Supplementary Figure 9 Quantitative real-time PCR (qRT-PCR) validation the potential target genes of TFs in different cold treatments. FPKM values (left) and relative expression (right) of DEGs in different cold times.

Supplementary Figure 10 Model for the dynamic changes of the transcriptome in short- and long-term cold treatments.
