# Supplementary figures and images for: Dynamic changes in the transcriptome landscape of Arabidopsis thaliana in response to cold stress
Source: Front Plant Sci. 2022 Aug 30;13:983460. doi: 10.3389/fpls.2022.983460 (PMC9468617; doi:10.3389/fpls.2022.983460)

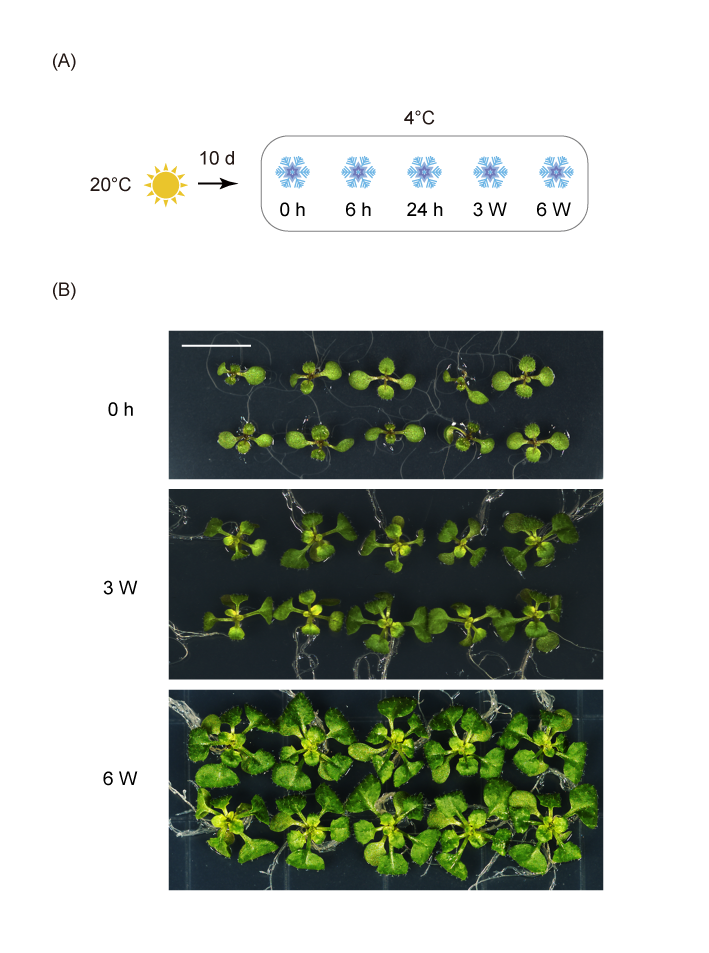

Supplement: Supplementary Figure 1 — Sample processing overview. (A) Time points of the sample are marked by the different labels, including 0 hour (0 h), 6 hours (6 h), 24 hours (24 h), 3 weeks (3 W), and 6 weeks (6 W). (B) Phenotypes of FRI-Col0 at 4°C for 0 h, 3 weeks (3 W), and 6 weeks (6 W). Bars = 1cm. [file Image_1.TIF]

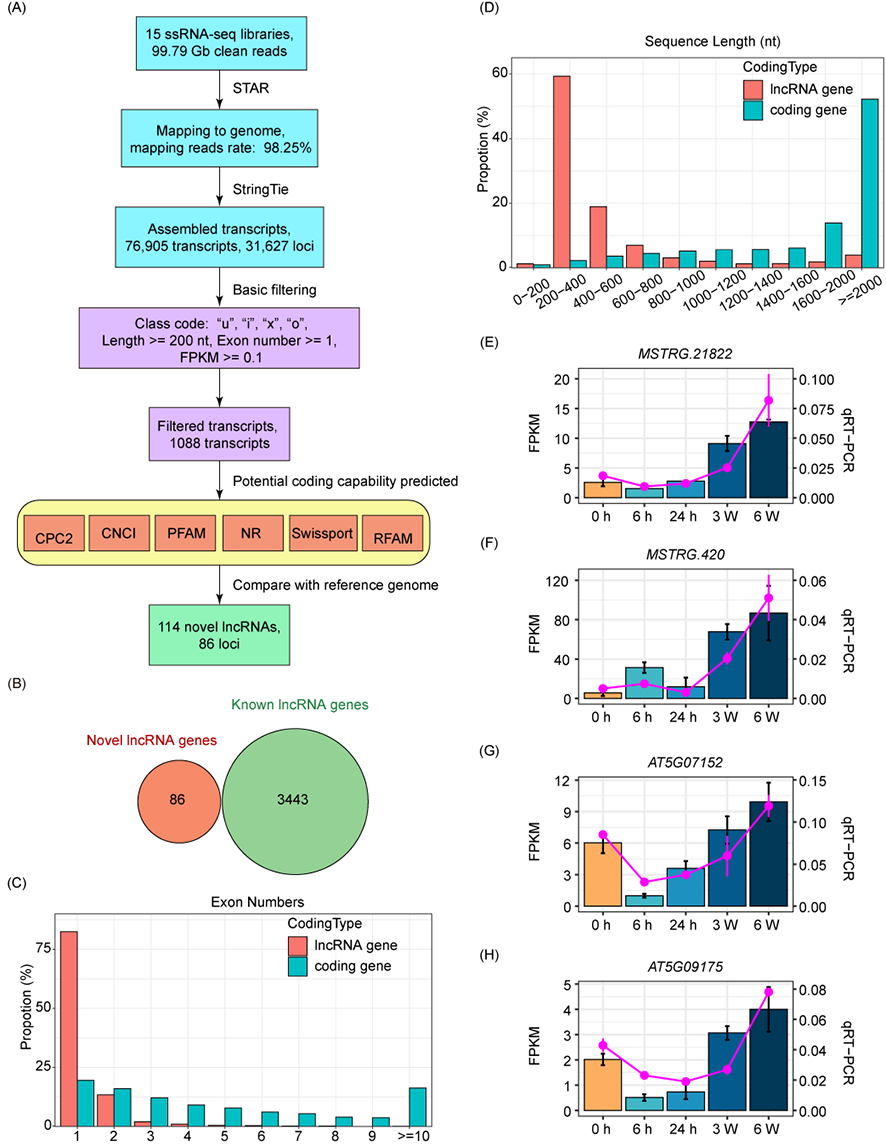

Supplement: Supplementary Figure 2 — Genome-wide identification of lncRNAs in Arabidopsis thaliana. (A) Pipeline for identification of lncRNAs. (B) Venn diagram showing the number of novel lncRNA genes and known lncRNA genes. (C) Distribution of exon numbers of lncRNA genes and coding genes. (D) Distribution of sequence length of lncRNA genes and coding genes. (E–H) Quantitative real-time PCR (qRT-PCR) validation of lncRNAs in different cold treatments. FPKM values (left) and relative expression (right) of lncRNAs in different cold times. [file Image_2.TIF]

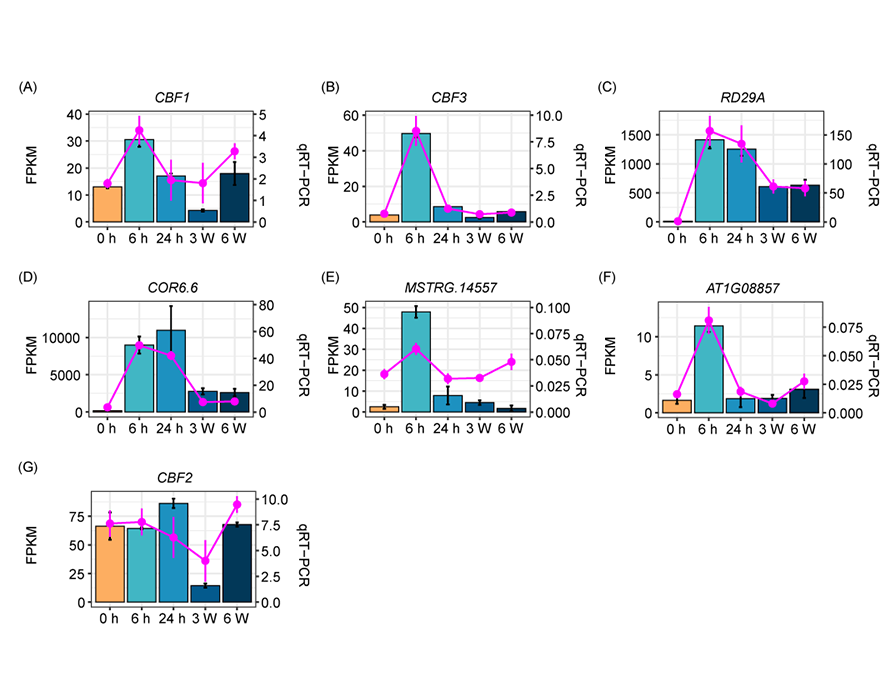

Supplement: Supplementary Figure 3 — Quantitative real-time PCR (qRT-PCR) validation the DEGs of short-term cold treatments of ssRNA-seq. FPKM values (left) and relative expression (right) of DEGs in different cold times. [file Image_3.TIF]

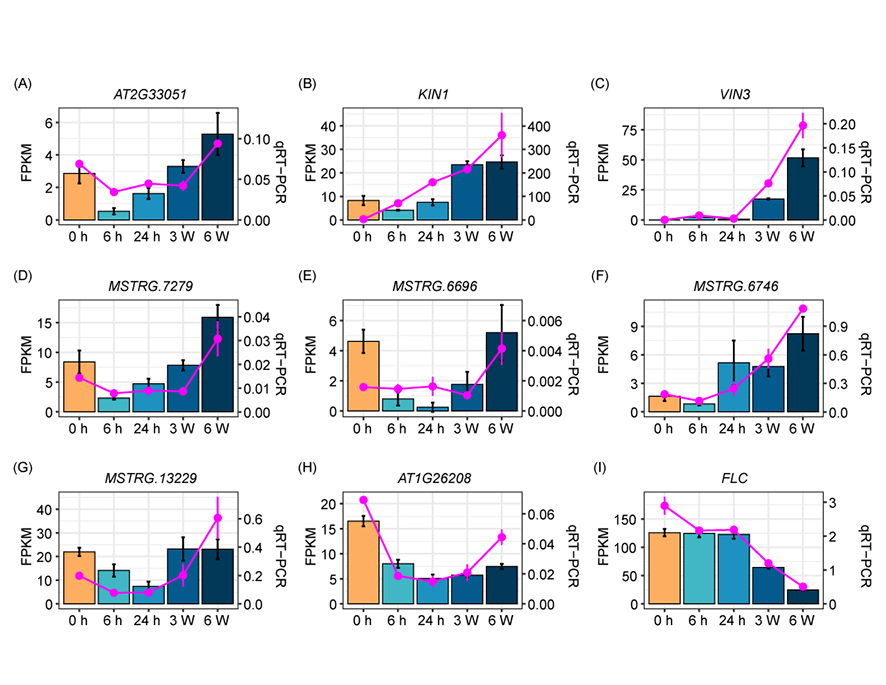

Supplement: Supplementary Figure 4 — Quantitative real-time PCR (qRT-PCR) validation the DEGs of long-term cold treatments of ssRNA-seq. FPKM values (left) and relative expression (right) of DEGs in different cold times. [file Image_4.TIF]

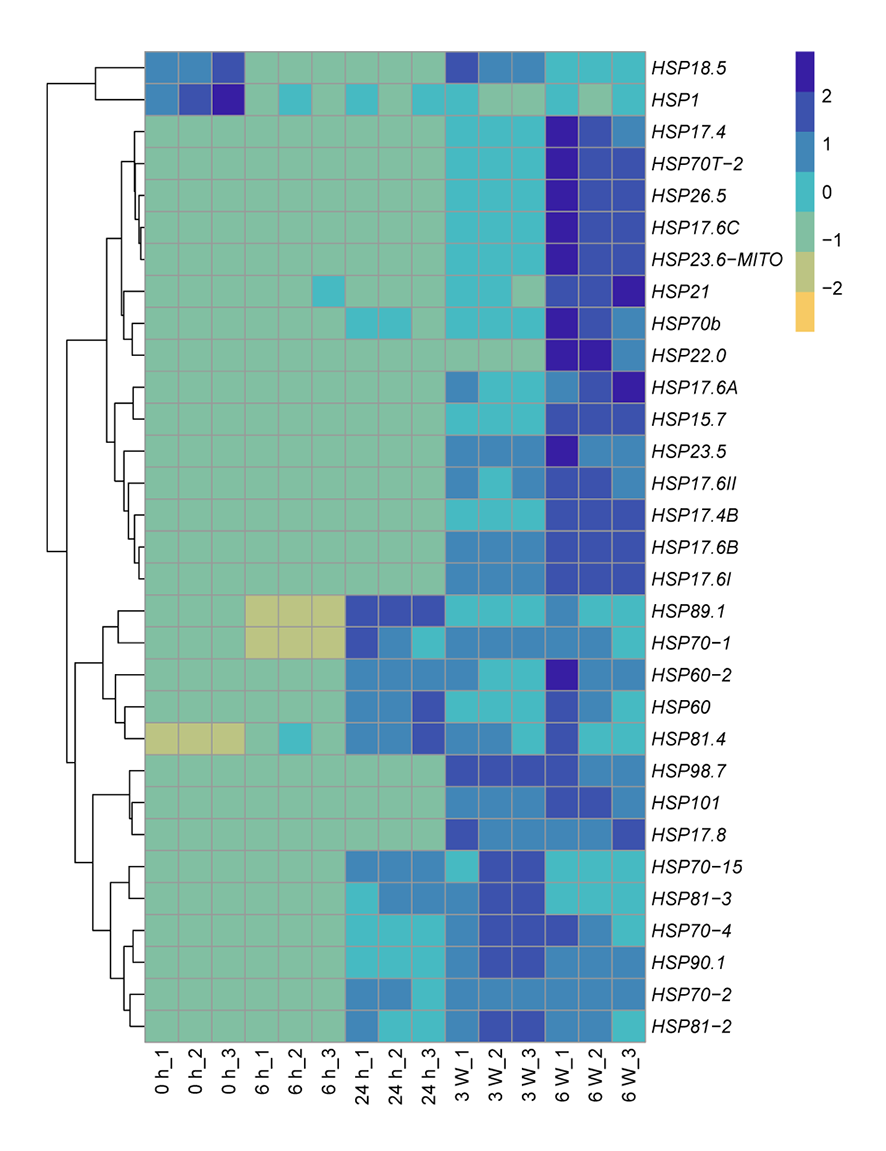

Supplement: Supplementary Figure 5 — Transcriptional profile of differentially expressed the heat shock proteins (HSP) genes in different cold treatment. The data are represented as a heatmap generated with scale = row dependent on FPKM. The color scale is shown on the right. [file Image_5.TIF]

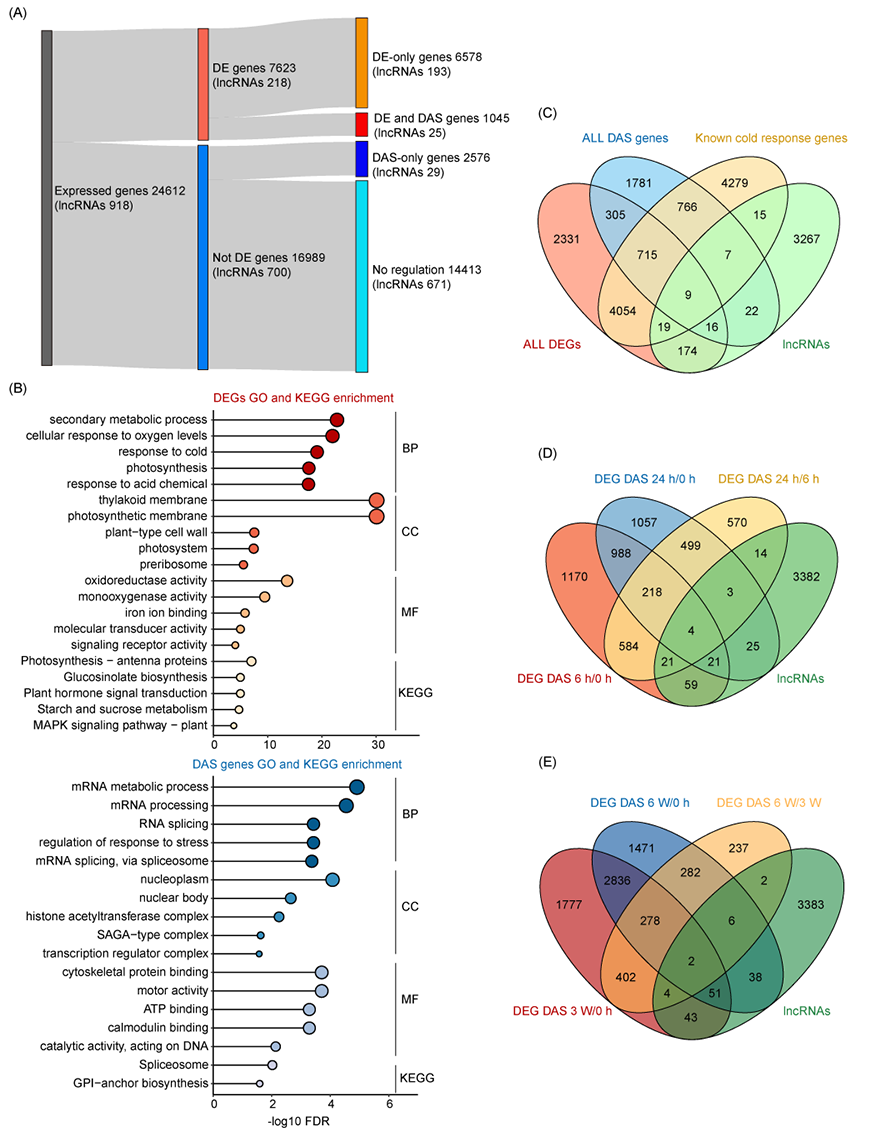

Supplement: Supplementary Figure 6 — DEG and DAS Analysis of Arabidopsis transcriptome during cold treatment. (A) Flow chart showing the distribution of the DEGs and DAS genes. (B) Most significantly enriched GO terms for DEGs and DAS genes. Bar plot of –log10 transformed FDR values are shown. (C) Venn diagram showing the DEGs and DAS genes identified and compared with known cold response DEGs and lncRNAs. (D) Venn diagram showing the DEGs and DAS genes in short-term cold treatment and compared with lncRNAs. (E) Venn diagram showing the DEG and DAS genes in long-term cold treatments and compared with lncRNAs. [file Image_6.TIF]

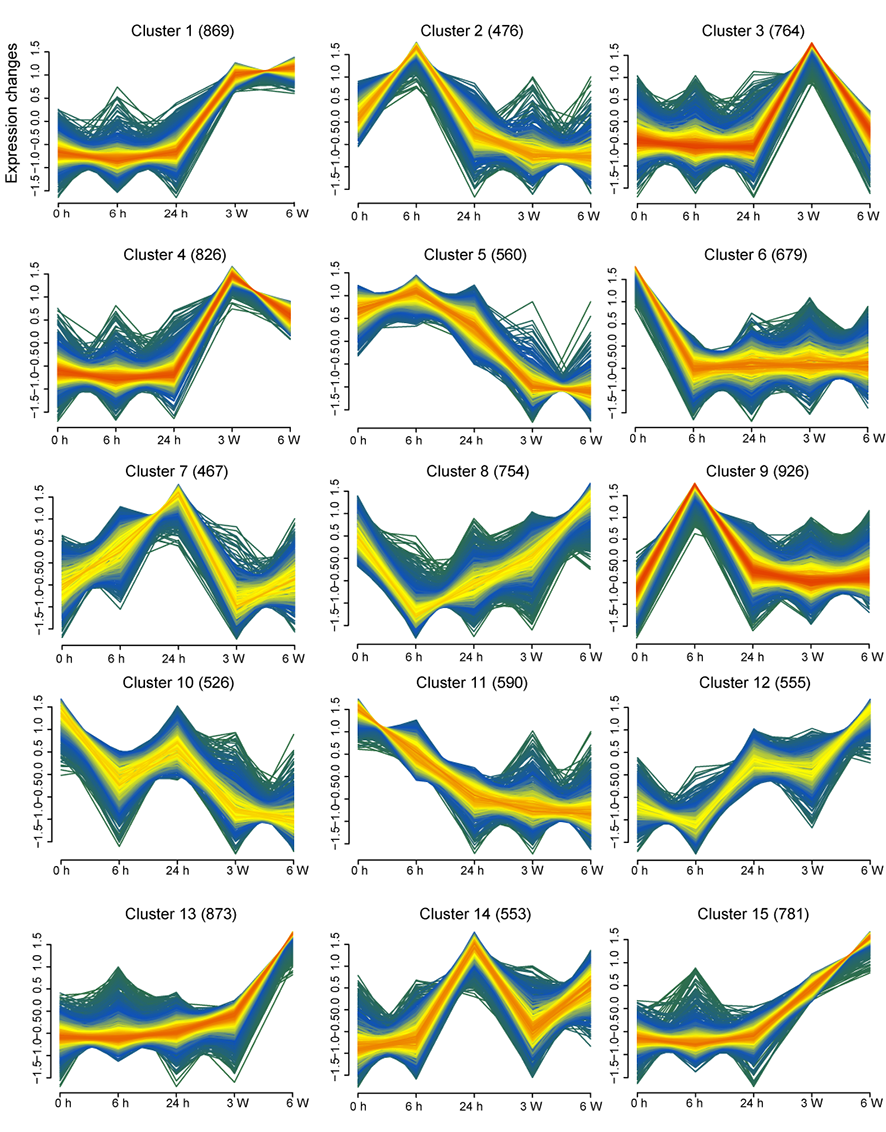

Supplement: Supplementary Figure 7 — Temporal expression dynamics were analyzed by standard-normal FPKM using the Mfuzz. Expression dynamic profiles were labeled 1-15. Each plot shows the profile for each cluster with high membership in warm colors (yellow, red), and low membership values in cool colors (green, blue). [file Image_7.TIF]

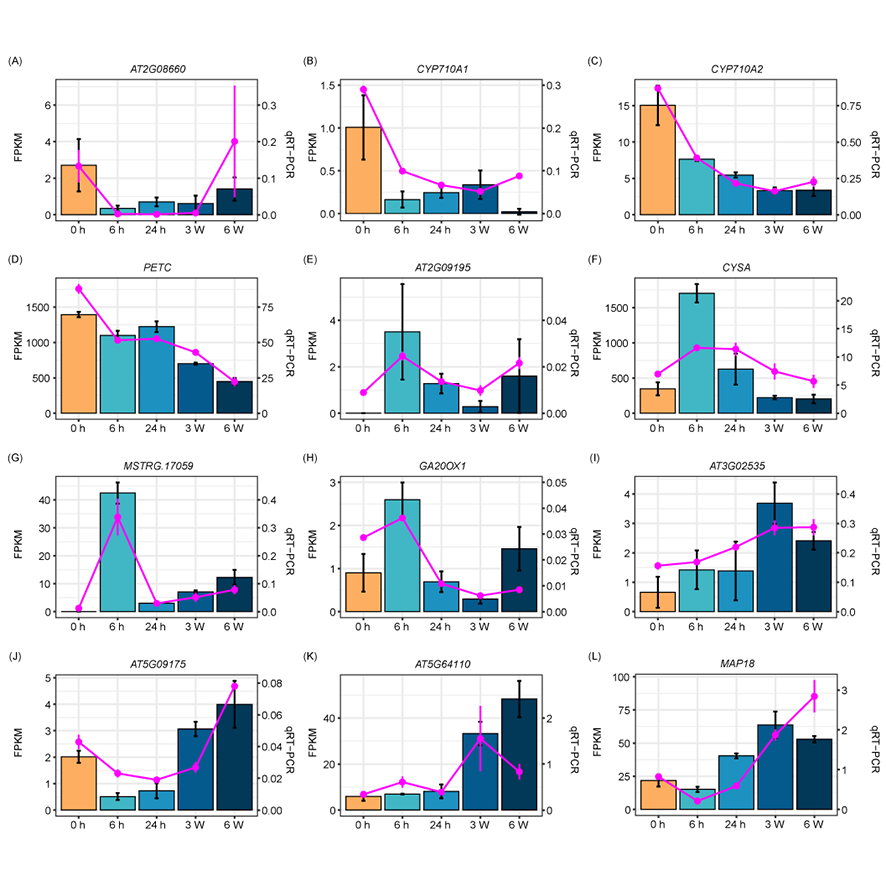

Supplement: Supplementary Figure 8 — Quantitative real-time PCR (qRT-PCR) validation the potential target genes of lncRNAs in different cold treatments. FPKM values (left) and relative expression (right) of DEGs in different cold times. [file Image_8.TIF]

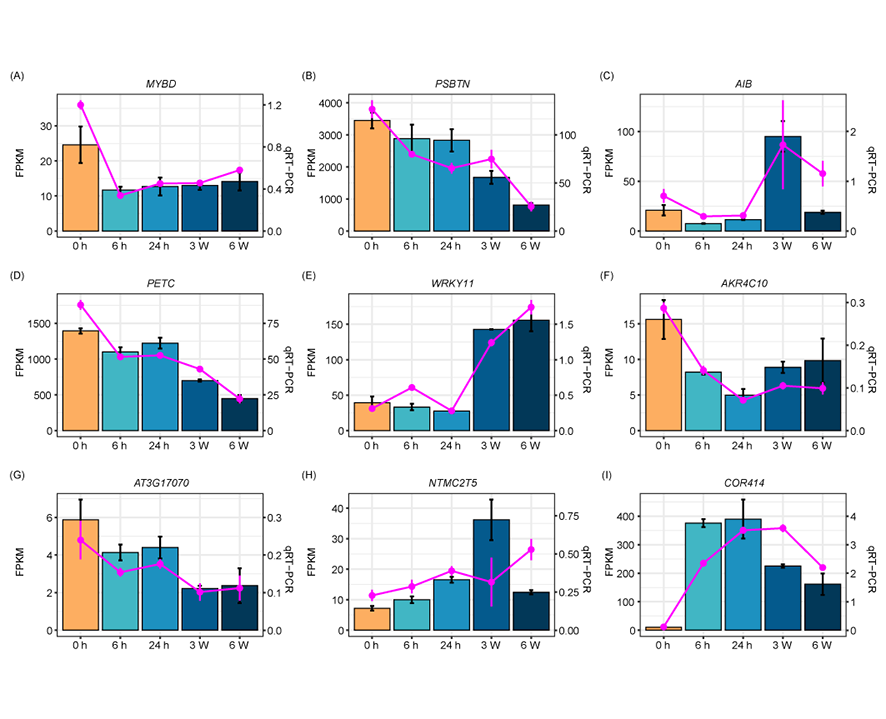

Supplement: Supplementary Figure 9 — Quantitative real-time PCR (qRT-PCR) validation the potential target genes of TFs in different cold treatments. FPKM values (left) and relative expression (right) of DEGs in different cold times. [file Image_9.TIF]

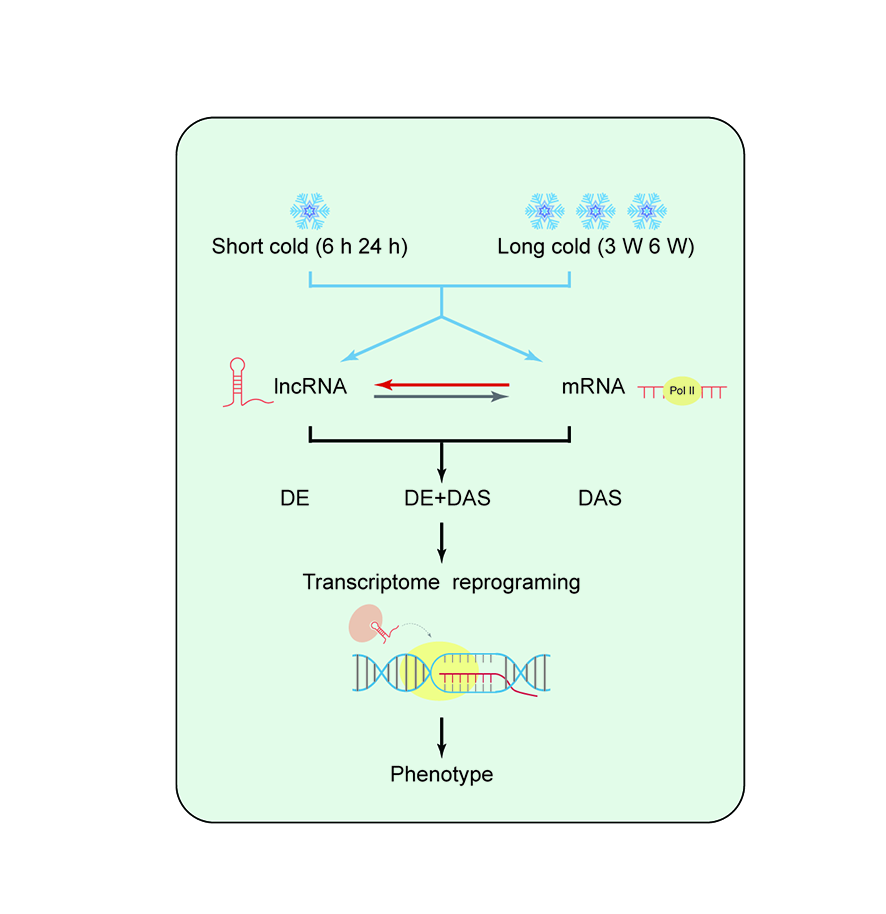

Supplement: Supplementary Figure 10 — Model for the dynamic changes of the transcriptome in short- and long-term cold treatments. [file Image_10.TIF]
